# The protein kinase CK1: Inhibition, activation, and possible allosteric modulation

**DOI:** 10.3389/fmolb.2022.916232

**Published:** 2022-08-24

**Authors:** Yashoda Krishna Sunkari, Laurent Meijer, Marc Flajolet

**Affiliations:** ^1^ Laboratory of Molecular and Cellular Neuroscience, The Rockefeller University, New York, NY, United States; ^2^ Perha Pharmaceuticals, Hôtel de Recherche, Roscoff, France

**Keywords:** casein kinase 1 (CK1), kinase inhibitor, allosteric, Alzheimer’s disease (AD), Neurodegeneration, DNA-encoded library (DEL)

## Abstract

Protein kinases play a vital role in biology and deregulation of kinases is implicated in numerous diseases ranging from cancer to neurodegenerative diseases, making them a major target class for the pharmaceutical industry. However, the high degree of conservation that exists between ATP-binding sites among kinases makes it difficult for current inhibitors to be highly specific. In the context of neurodegeneration, several groups including ours, have linked different kinases such as CK1 and Alzheimer’s disease for example. Strictly CK1-isoform specific regulators do not exist and known CK1 inhibitors are inhibiting the enzymatic activity, targeting the ATP-binding site. Here we review compounds known to target CK1, as well as other inhibitory types that could benefit CK1. We introduce the DNA-encoded library (DEL) technology that might represent an interesting approach to uncover allosteric modulators instead of ATP competitors. Such a strategy, taking into account known allosteric inhibitors and mechanisms, might help designing modulators that are more specific towards a specific kinase, and in the case of CK1, toward specific isoforms.

## Background

Improvements in disease diagnostics, scientific progress in therapeutic target identification, and advances in highly specific and safe therapeutic drugs represent relentless engines to address the need of new medicinal treatments, especially in the field of neurodegeneration such as Alzheimer’s disease. Behind the scenes, in the back end of the pharmaceutical world, drug screening represents the backbone of a very active industry focusing on identifying new drug-like molecules. While, new treatments are certainly needed, novel therapeutic strategies and tools are also needed.

Protein kinases play a vital role in biology by catalyzing the transfer of a phosphate group, onto protein substrates, at the hydroxy position of specific amino acids, modifying their activity or function (Johnson and Lewis, 2001; Ardito et al., 2017). Deregulation of kinases and their substrates is implicated in numerous diseases, and for that reason alone kinases represent a major target class for developing novel therapeutics (Zhou et al., 2010; Rebholz et al., 2013; Roskoski, 2015; Yuan et al., 2019; Cohen et al., 2021; De Pins et al., 2021; Roskoski, 2021). In the context of neurodegeneration, and for Alzheimer’s disease in particular, a number of protein kinases have been involved with relevance to different key pathways (Flajolet et al., 2007; Bustos et al., 2017a; Bustos et al., 2017b). For example, tau hyper-phosphorylation is directly linked to tau pathology and a great deal of effort is being devoted to reduce tau hyperphosphorylation (Rubenstein et al., 2019; Wakeman et al., 2022); for a recent review see (Wegmann et al., 2021). About 30 phosphorylation sites have been found to be phosphorylated only in normal brain samples and the specific nature of the sites involved might be less important than the overall level of phosphorylation, the overall net charge of tau. Therefore, blocking or reducing the kinase activity of a kinase such as CK1 potentially targeting numerous sites, directly and indirectly *via* kinase priming, could be highly beneficial.

## Casein kinase 1 (CK1) isoforms are involved in diseases and disorders

The role of CK1 in numerous diseases ranging from host-parasite interactions, to highly complex diseases such as cancer and neurodegeneration, has been well established (Knippschild et al., 2005b; Flajolet et al., 2007; Zhou et al., 2010; Schittek and Sinnberg, 2014; Bustos et al., 2017a; Bustos et al., 2017b; Chen et al., 2017; Zhou et al., 2020; Rachidi et al., 2021). Strictly CK1-isoform specific regulators have not been described (Qiao et al., 2019; Du et al., 2021), and most CK1 small molecular weight regulators are inhibiting the enzymatic activity (Li et al., 2021). Only a limited number of CK1 modulators have been fully validated and none of the currently known CK1 inhibitors have reached clinical stage (at the exception of PF05251749 that may enter clinical evaluation in the near future).

CK1, one of the very first identified protein kinases, is ubiquitously expressed and defines a family of serine/threonine kinases, highly conserved in all eukaryotic organisms (Knippschild et al., 2005a). In mammalians, seven CK1 isoforms ( α, β, γ1, γ2, γ3, δ and ε) with various splice variants have been characterized. Little is known about the regulation of CK1 *in-vivo*. It is believed that CK1 is basally active and at least some isoforms (e.g., CK1δ and CK1ε) are regulated by inhibitory autophosphorylation at their C-terminal regions. Importantly, the various isoforms are not equally distributed and their regionalized expression in various organs makes it an attractive target for disorders and diseases. In the brain, isoforms might be expressed in different neuronal and non-neuronal populations, localized in various regions (e.g., cellular membrane vs. cytoplasm; cytoplasm vs. nucleus) of the cell presenting different pools of substrates. Importantly, not all isoforms are equally distributed and expressed in different organs, and the existence of CK1 brain specific isoforms makes it an attractive target class for brain disorders and diseases.

In the context of neuronal dysfunctions, CK1 has been abundantly linked to the regulation of the dopaminergic pathway with repercussions on the action of psychostimulants and ADHD (Zhou et al., 2010; Zhou et al., 2020) among others. CK1 is also highly relevant for neurodegeneration, and especially Alzheimer’s disease (AD) (Flajolet et al., 2007). Several studies, from our group and others, have linked CK1 and AD, both in relation to amyloid-Abeta peptide and the protein tau (Walter et al., 1998; Schwab et al., 2000; Yasojima et al., 2000; Pastorino et al., 2002; Flajolet et al., 2007). Briefly, after realizing that CK1 expression was increased in AD brains, we demonstrated that overexpression of CK1 ε increased amyloid-Abeta peptide production. Furthermore, three CK1 inhibitors (Figure 2), compounds **1–3**) significantly reduced endogenous Abeta peptide production in cultured cells without affecting Notch cleavage (Flajolet et al., 2007).

CK1 might act directly by phosphorylating a target as reviewed above but it can also act indirectly via other kinases by activating or priming them, such as GSK3 for which CK1 is a priming kinase together with CK2, CDK5, DYRK1A and PKA (Meijer et al., 2004). CK1 also has the potential to act as an upstream regulator of CDK5 which is also implicated in AD (Liu et al., 2001; Walter et al., 2001). In addition, CK1δ is regulated by CDK2 and CDK5 (Ianes et al., 2016).

In summary, the presence of numerous CK1 consensus sites on important therapeutic targets, the existence of organ and tissue regionality of CK1 isoforms, and the possibility that CK1 can prime other important kinases such as GSK3 β , give CK1 family a significant advantage to meaningfully affect important diseases and disorders.

## Casein kinase 1 inhibitors target the ATP-binding site

**FIGURE 1:**
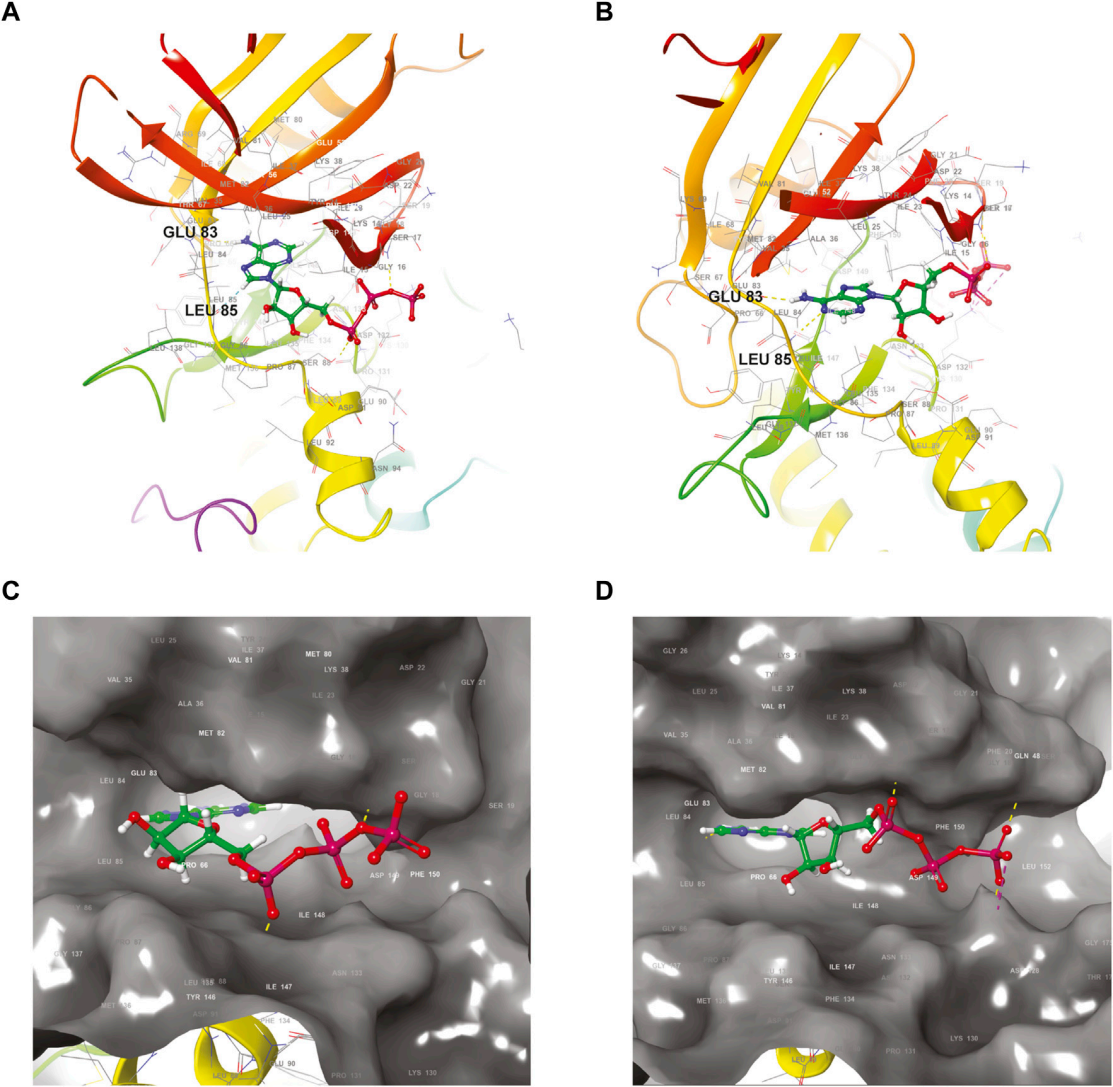
Structural comparison of the ATP-binding site of the two closely related CK1 isoforms delta and epsilon. **(A,B)** Ribbon diagrams of the polypeptidic backbones of CK1 delta **(A)** (PDB accession code 6RCG) and CK1 epsilon **(B)** (PDB accession code 4HNI). Ball-and-stick representations of an ATP molecule docked in the active site of CK1 delta **(C)** and CK1 epsilon **(D)** shown as surface models. The ATP tri-phosphate moieties are highlighted in red. PDB: Protein Data bank.

Current CK1 inhibitors are targeting the ATP-binding site and functioning as ATP-competitive molecules. The ATP-binding site is the most conserved site in protein kinases and even more so among homologous family members. The ATP-binding site or orthosteric site is a well-defined pocket which forms a rigid and deep binding cavity. It represents an ideal structure for drug-like molecules aiming at ATP-competitive inhibition (Gray et al., 1999; Ardito et al., 2017). For these reasons, the ATP-binding site represents a highly coveted target option for kinase inhibition even though challenging (Ayala-Aguilera et al., 2022). However, inhibitors targeting a specific ATP-binding site can easily bind to other ATP-dependent enzymes, including kinases, and especially homologous family members. In the case of two brain isoforms, CK1 δ and CK1 ε , it is difficult to target specifically the ATP-binding site of just one isoform due to their highly conserved sequence as demonstrated in Figure 1 (Du et al., 2021). The adenine region involves GLU 83 and LEU 85 in both cases (Figures 1A,B) and is well conserved among CK1 isoforms in general. The overall ATP molecule is positioned very similarly in both isoforms (Figures 1C,D). Any undesired off-targeting activity can be associated with higher side-effects and deleterious clinical consequences.

Kinases adopt either a structurally active (DFG-in) or inactive (DFG-out) state conformation for their binding state and nearly all described kinase inhibitors are based on one of these two conformational configurations, which are also known as Type I and type II respectively (Kanev et al., 2019; Attwood et al., 2021).

**FIGURE 2:**
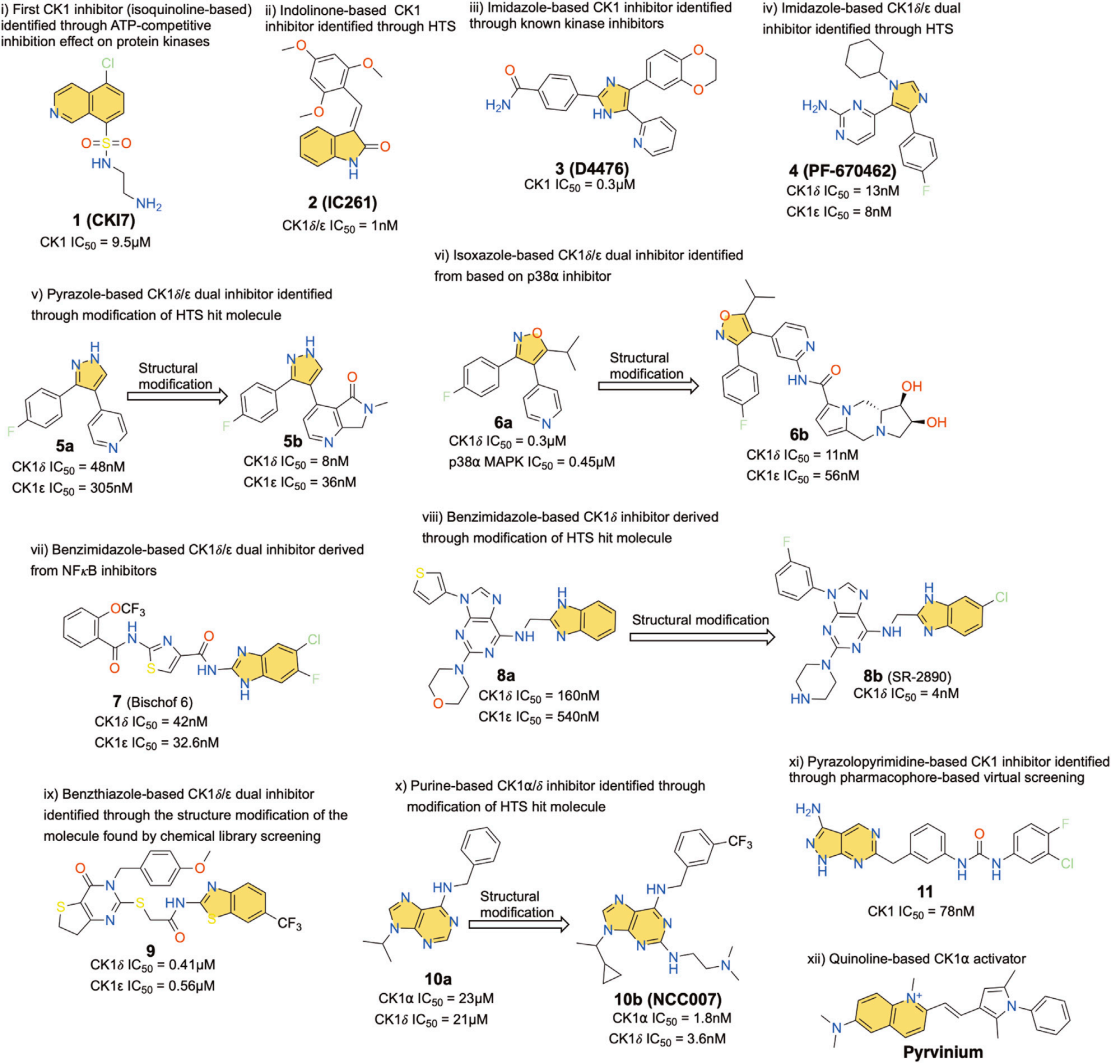
Representative chemical structures displaying a kinase inhibitory activity and their potency on CK1 family members. Yellow indicates various priviledged chemo-motifs. Heteroatoms are highlighted with standard colors.

CK1 inhibitors described in the literature can be grouped into various classes based on their basic chemical scaffold. The different compound types are presented in Figure 2. For each scaffold type, the most active compound has been selected as a representative member of the family. These compounds were identified over the last 25 years using various technologies ranging from high-throughput screening (HTS) to fragment-based drug discovery and structure-based virtual screening (Du et al., 2021; Li et al., 2021). The IC_50_ of each compound is also indicated and corresponds to different types of assays and systems, mostly obtained from *in-vitro* studies.

Compound **1**, well known as CKI7, is an isoquinoline-based derivative, and it was the first CK1 inhibitor identified historically (Xu et al., 1996). Compound **2**, or IC261, another early stage CK1 inhibitor, is an indolinone-based derivative, and it was found as a dual CK1δ/ε   specific inhibitor through HTS (Mashhoon et al., 2000). Compounds **3** (D4476) and **4** (PF-670462) are imidazole-based molecules which were derived from known kinase inhibitors and HTS respectively (Badura et al., 2007; Cheong et al., 2015). The inhibitory effect of compound **3** does not present any degree of specificity toward CK1 isoforms (perhaps slightly CK1 δ ), it also targets other kinases such as P38 α and PDK1. Inhibitor four at the contrary is CK1δ/ε specific and is active in the low nanomolar range. Compound **5b** is a pyrazole-based molecule identified by modifications of hit molecule found through HTS as CK1δ/ε inhibitors with an IC_50_ of 8 and 36nM, respectively (Wager et al., 2017a; Wager et al., 2017b). Compound **6b** is an isoxazole-based derivative that also inhibits both CK1δ/ε in the nanomolar range; this compound was derived from a p38α kinase inhibitor (Peifer et al., 2009; Luxenburger et al., 2019). Compound **7** and **8a,b** are benzimidazole-based compounds. Compound **7** is a dual CK1δ/ε inhibitor that was derived from an NF κ B inhibitor, whereas hit molecule **8a** was obtained by HTS and used after structural modifications to derive **8b** (Bischof et al., 2012; Bibian et al., 2013). Of note, **8b** also inhibits six other kinases (>90%) out of a panel of 442 kinases tested (Bischof et al., 2012). Compound **9**, a benzothiazole derivative, was identified as a dual CK1δ/ε inhibitor and resulted from structural modification of an initial hit molecule identified from low throughout screening (Salado et al., 2014; Garcia-Reyes et al., 2018). Compound **10a**, a purine-based derivative, was identified by HTS as a dual CK1 α/δ and structure activity relationship-based optimization led to a very potent inhibitor **10b** (Lee et al., 2019). Compound **11** is a pyrazolopyrimidine-based scaffold and was identified from pharmacophore-based screening. It inhibits CK1 (nanomolar range) but does not present isoform-specific inhibition (Yang et al., 2012).

In addition to selectivity related issues, there is some resistance to the development of current inhibitors targeting the ATP-binding site and its immediate vicinity including the gate keeper position(s) (Lovly and Shaw, 2014; Kim et al., 2021). Several studies demonstrated a higher mutagenicity of those regions in response to chemotherapies. Such mutations, especially frequent in various types of cancers, lead quickly to treatment resistance by by-passing classical signaling pathways. For example, the Receptor Tyrosine Kinase (RTK) EGFR (Epidermal Growth Factor Receptor) is a key regulator for important biological processes. Abnormal expression of this receptor is associated with a wide range of cancer types. FDA-approved drugs targeting the kinase activity are available and they mostly target the ATP-competitive orthosteric site. Resistance is often observed due to mutations in the vicinity of the ATP-binding site. Furthermore, the rigid and conserved nature of this orthosteric binding region makes drug design more complicated in general, and even more so when isoform specificity is required.

## CK1 activator

It is legendary difficult to develop kinase activators, and in many cases, it might even be impossible. Interestingly, one CK1 activator has been reported. Indeed, known kinase activators are extremely rare and not always identified based on a rational design. Kinases can be constitutively active, activated by another protein (e.g., cyclins for CDKs), activated upon intracellular relocalization or modified by alteration of their post-translational modification profile. None of these properties are particularly easy to manipulate with drug-like compounds.

Interestingly, pyrvinium, a reported CK1 activator is believed to activate CK1 α, and leading to Wnt signaling pathway inhibition (Thorne et al., 2010) [Figure 2 (xii)]. Although several reports mentioned pyrvinium as a CK1 α activator, the mechanism underlying this activation remains controversial. A recent detailed biochemical analysis by Chen et al. supports that pyrvinium enhances CK1 α kinase activity through allosteric modulation (Shen et al., 2019).

## Successful allosteric kinase inhibitors

**FIGURE 3:**
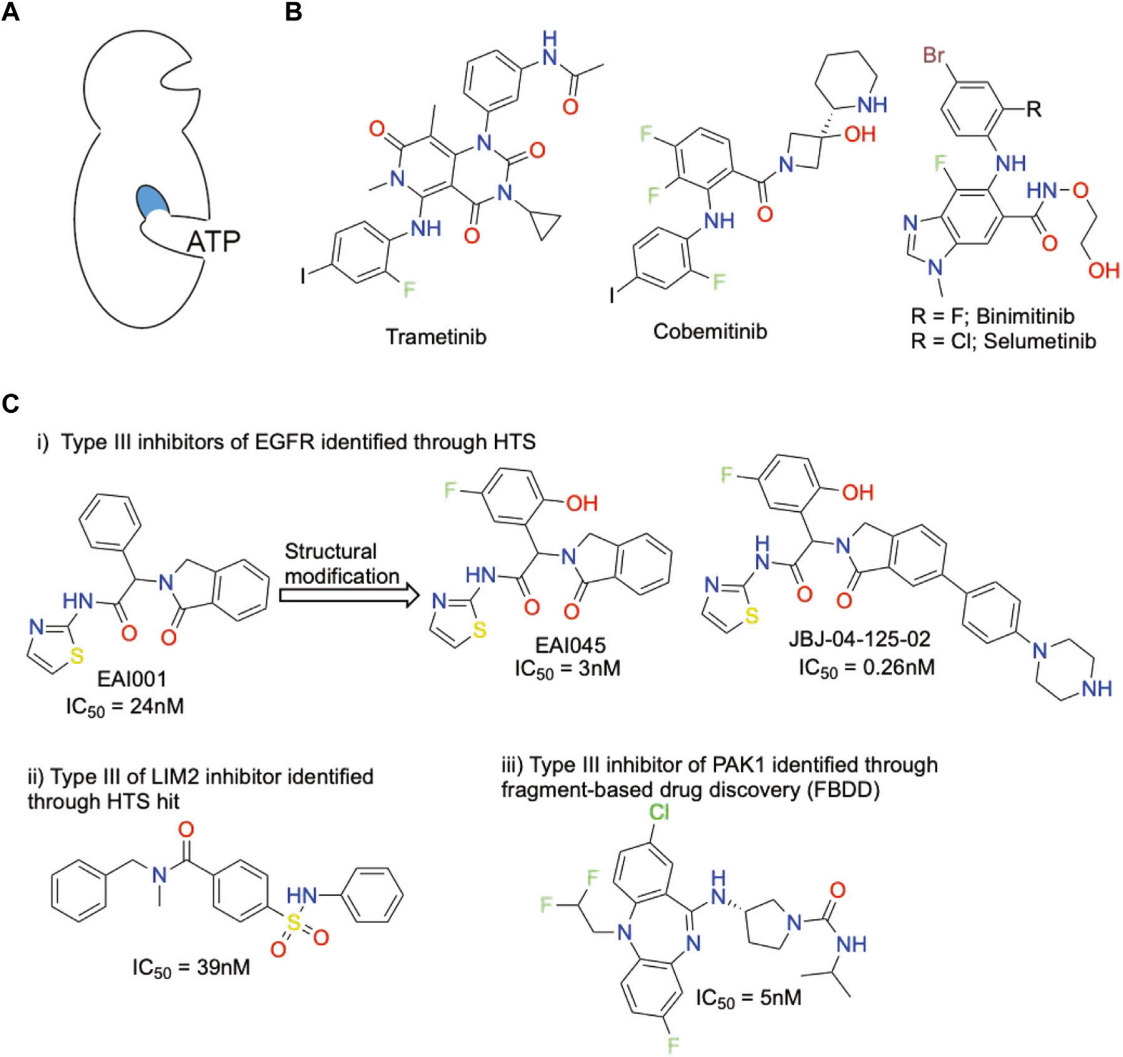
Representative chemical structures displaying a kinase inhibitory activity of Type III and their potency toward specific kinases. **(A)** Schematic representation of Type III allosteric site. Blue color indicates targetted site. **(B)** FDA-approved Type III HEK inhibitors **(C)** Type III kinase inhibitors.

**FIGURE 4:**
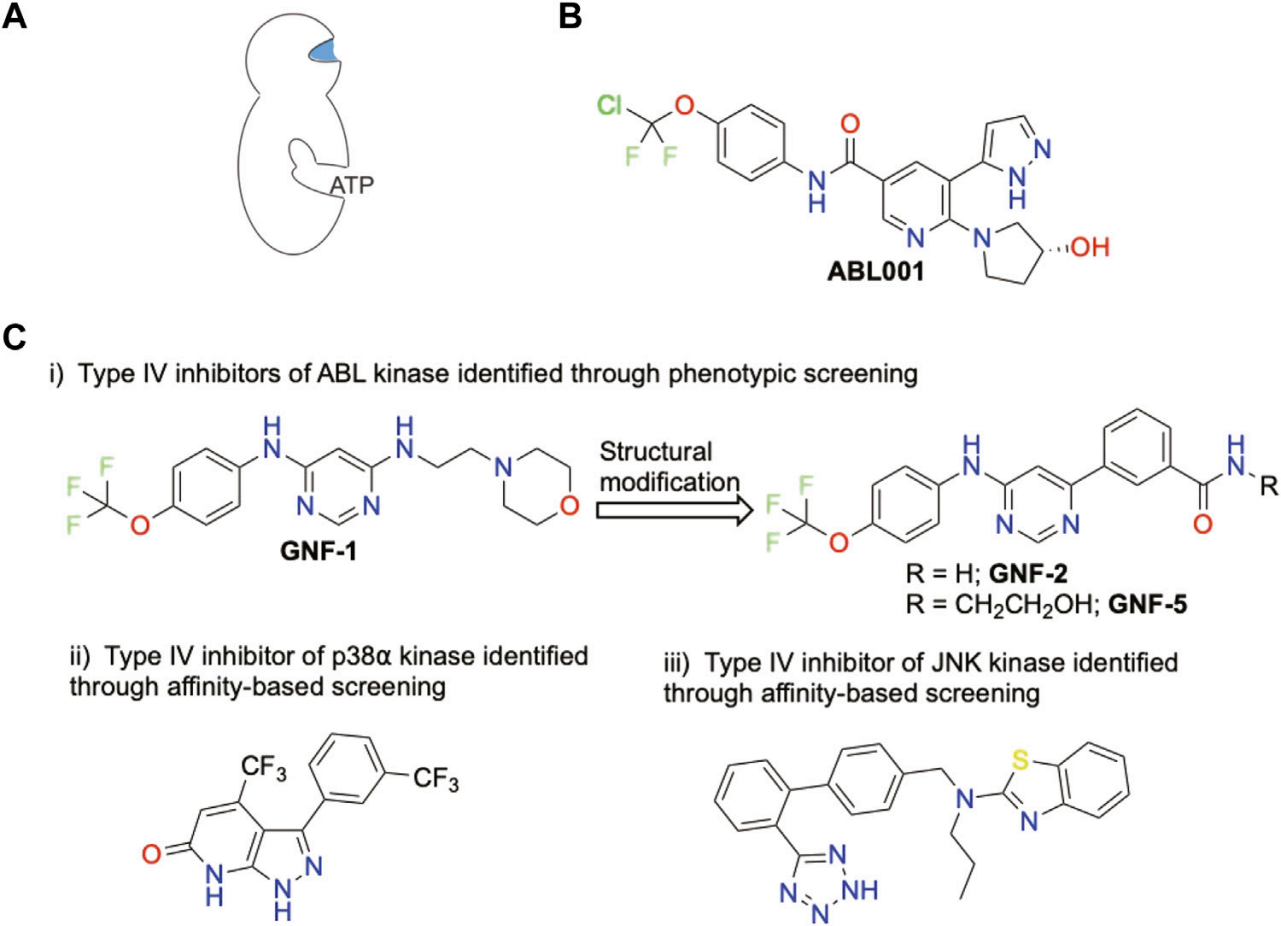
Representative chemical structures displaying a kinase inhibitory activity of Type IV and their potency toward specific kinases. **(A)** Schematic representation of Type IV allosteric site. Blue color indicates targetted site. **(B)** Only FDA-approved Type IV inhibitor **(C)** Other Type IV inhibitors

Allosteric sites are crucial to regulate the activity of a biological target in a dynamic way requiring an inducible structural rearrangement. A drug-like compound acting as a CK1 allosteric inhibitor could be promising for several medical applications. Allosteric sites are less constraint, present a larger diversity with reduced off-target issues, resulting in an increased selectivity and/or specificity. Identifying putative allosteric sites is challenging including when benefiting from the existence of a crystal structure. A co-crystal structure with a specific ligand uncovering the allosteric site is ideal but not simple to obtain. Cryptic pockets are rarely visible by definition, unless a specific conformation of the protein target is induced, either chemically or biologically. Allosteric modulators are classified as Type III and Type IV allosteric regulators based on the distance from the ATP-binding domain of the kinase domain. Type III modulators bind to a site adjacent to the ATP-binding site and Type IV to a site located further away (see schematic representations Figures 3A, 4A).

Despite the difficulties, several kinase allosteric modulators were successfully developed and even approved for clinical use by the FDA, clearly demonstrating the value and feasibility of identifying kinase allosteric modulators. Trametinib is the first non-ATP-competitive (allosteric) FDA-approved drug that was identified as MEK inhibitor in 2013 (Wright and Mccormack, 2013). MEK, a member of the mitogen-activated protein kinase family, is a dual specificity threonine/tyrosine kinase that plays a critical role in the Ras - Raf (MAPKKK) - MEK1/2 (MAPKK) - ERK1/2 (MAPK) signaling pathway. Cobemitinib (Garnock-Jones, 2015), Binimitinib (Rose, 2019) and Selumetinib (Casey et al., 2021) were three others FDA-approved Type III allosteric inhibitors for MEK1/2 (Figure 3B) and others are in clinical development (Shang et al., 2016).

Those FDA-approved allosteric drugs were identified using different technologies mostly centered on HTS and taking advantage of cellular or biochemical assays. Besides MEK, the most allosterically studied kinase is AKT and some of AKT allosteric regulators are currently being tested in clinical trials (Revathidevi and Munirajan, 2019; Martorana et al., 2021).

Compound EAI001 [Figure 3C (i)] was identified as Type III allosteric inhibitor by HTS and structural modifications led to more potent inhibitors (EAI045 and JBJ-04-125-02) (Jia et al., 2016; To et al., 2019). Sulfonamide-derivative was identified as LIMK2 Type III allosteric inhibitor through HTS hit molecule [Figure 3C (ii)] and this derivative has good selectivity over its closely related kinase LIMK1 (Goodwin et al., 2015). Based on fragment-based drug discovery a dibenzodiazepine compound was derived as Type III allosteric inhibitor for PAK1 kinase (Karpov et al., 2015) [Figure 2C (iii)]. Type III allosteric inhibitors were also reported for the kinases IRE1 and RIP1 (Wu et al., 2015).

Finally, type IV allosteric inhibitors correspond to molecules which bind a distal allosteric site from the ATP-binding site (Figure 4A). At this date, Asciminib (ABL001) is the only FDA-approved Type IV inhibitor (Deeks, 2022) (Figure 4B). ABL001 was identified initially through phenotypic screening targeting ABL kinase and further optimized by fragment-based NMR (Wylie et al., 2017). Type IV modulators are typically identified by conventional technologies such as phenotypic screening (Adrian et al., 2006) and affinity-based screenings (Comess et al., 2011) (Figure 4C).

Recently, Lu et al. categorized two other types of kinase allosteric regulators that bind outside the kinase domain. Type VI binds to the catalytic inactive site of a pseudokinase domain while Type VII binds elsewhere outside the kinase domain, for example in the extracellular region of a receptor tyrosine kinase (Lu et al., 2020).

The absence of CK1 allosteric inhibitors probably results from the convergence of the high degree of conservation between isoforms and the absence of an assay compatible with high throughput allosteric testing. Regarding the degree of similarity between isoforms, differences do exist in the primary sequences and those could allow drug-like molecules to bind with some degree of specificity. Regarding the screening assay, the DNA-encoded library (DEL) technology does not require a functional assay. Indeed, DEL offers the possibility to search for molecules binding to the target very efficiently (hundreds of millions to billions of compounds). Allosteric regulation of the binding molecules can then be evaluated as a validation step or secondary assay, in a low throughput and well controlled enzymatic assay. Furthermore, the possibility to perform DEL screens in parallel (e.g., two or more isoforms, or wild type versus mutant) allows early on in the process, at the very first step, to select specific binders.

## DNA-encoded libraries and non-biased approaches to tackle allostery

For the reasons mentioned previously, alternative therapeutic strategies directed towards the development of molecules targeting non-ATP binding sites have emerged and are becoming more popular (Wu et al., 2015; Lu et al., 2019; Lu et al., 2020). The DEL technology might be particularly adapted to identify allosteric modulators and to target cryptic pockets in general, due to its screening concept that is based solely on binding affinity (Schwab et al., 2000; Gironda-Martinez et al., 2021; Huang et al., 2022). With this approach, a very large number of compounds this was mentioned just before covalently tagged with a unique and specific DNA sequence are mixed with the immobilized protein target in a single tube. Following the incubation step, several washes are performed and at the end of the process, compounds binding to the target are identified through PCR amplification and next generation sequencing. The DNA tag sequences allow for the hit molecules to be identified. Validation of the hit binders can then be performed individually ON-DNA and OFF-DNA. Validated binders can then be investigated for their function.

This screening approach is totally independent of a specific activity, and therefore does not require any type of assay. Furthermore, due to the practicality of such screening campaigns, several conditions can be tested in parallel and subtractive approaches can be utilized. For example, an existing inhibitor known to fit in the active site could be added during the DEL screening incubation in order to saturate the active site and block its accessibility to DEL molecules, favoring the binding of DEL compounds at other sites. A wild-type kinase could be screened in parallel to a dead kinase mutant, a single target protein could be screened in parallel to the same kinase associated to a co-factor if folding is known to be affected, a desired isoform could be compared to a non-desired isoform for specificity reasons, etc … DEL might prove useful to distinguish them in a non-biased approach. Using the DEL technology, kinase inhibitors and allosteric inhibitors for GPCRs have already been identified (Petersen et al., 2016; Ahn et al., 2017; Chang et al., 2017; Ahn et al., 2018; Kim et al., 2018; Kim et al., 2019; Guilinger et al., 2021). Finally, a validated compound binding to a target, even if ineffective at modulating the activity *per se*, could be used as a PROTAC anchor or alternatively as a biotracer or research tool.

## Conclusion

CK1 kinases regulate a wide variety of cellular processes and dysfunction of several members of this family have been involved in serious pathological conditions. In the context of AD, the multiple links connecting CK1 to various pathophysiological issues, its dual capacity to act on the two most important pathways (tau and amyloid beta), and the existence of well-defined neuronal specific isoforms, makes it a special and highly relevant target. We summarized here the compounds known to inhibit CK1 and for each privileged scaffold we present the most active compound as well as the method used to identify those compounds. All known CK1 inhibitors are targeting the ATP-binding site and the only activator known might do so via allosteric regulation. None of those drug-like molecules reached clinical stage. Due to the high degree of conservation between CK1 isoforms, and kinases in general, allosteric inhibitors might be more suited for therapeutic interventions. Identifying allosteric modulators is challenging but we believe that DNA-encoded library technologies, due to their absence of strong screening requirements and their unprecedented library size, are particularly well suited for the task.
